# Intervention for the Management of Neuropsychiatric Symptoms to Reduce Caregiver Stress: Protocol for the Mindful and Self-Compassion Care Intervention for Caregivers of Persons Living With Dementia

**DOI:** 10.2196/58356

**Published:** 2024-10-11

**Authors:** Aniyah Travis, Arden O’Donnell, Natalia Giraldo-Santiago, Sarah M Stone, Daniel Torres, Shelley R Adler, Ana-Maria Vranceanu, Christine S Ritchie

**Affiliations:** 1 Center for Aging and Serious Illness Massachusetts General Hospital Boston, MA United States; 2 Center for Health Outcomes and Interdisciplinary Research, Department of Psychiatry Massachusetts General Hospital Boston, MA United States; 3 Osher Center for Integrative Health University of California, San Francisco San Francisco, CA United States; 4 Harvard Medical School Boston, MA United States

**Keywords:** mindfulness, caregiver, self-compassion, ADRD, Alzheimer’s disease and related dementias, mental health

## Abstract

**Background:**

Stress related to Alzheimer disease and related dementias (ADRD) is common, particularly among those who care for persons with challenging behaviors and personality or mood changes. Mindfulness and self-compassion programs are efficacious for managing stress. The skills of mindfulness and self-compassion, however, must be integrated with behavioral management skills in order to effectively improve caregiver stress.

**Objective:**

In this study, we aimed to describe the development of the Mindful and Self-Compassionate Care (MASC) program, the first program that combines mindfulness and self-compassion with behavioral management skills to decrease caregiver stress, and its evaluation in the Supporting Our Caregivers in ADRD Learning (SOCIAL) study.

**Methods:**

Using the National Institutes of Health (NIH) stage model, we describe 3 phases of work encompassing NIH Stages 1A and 1B. In phase 1, we conducted 5 focus groups (N=28) of stressed individuals caring for persons with ADRD and challenging behaviors. Rapid data analysis informed the development of a 6-week online intervention. Phase 2 (NIH stage 1A) includes an open pilot (N>10) with optional exit interviews. Phase 3 (NIH stage 1B) is a feasibility randomized controlled trial of the intervention versus the Health Education Program control. Primary outcomes focus on feasibility with secondary outcomes encompassing acceptability, credibility, fidelity, and signals of preliminary efficacy. Phase 1 follows traditional recommendations for qualitative analyses (at the point of thematic saturation) which was achieved after 5 focus groups (N=28). For the phase 2 open pilot, up to 12 participants will be recruited. For the phase 3 feasibility study, recruitment of 80 caregivers will allow the assessment of feasibility benchmarks. Data for phase 1 included 5 focus groups. In phases 2 and 3, data collection will occur through REDCap (Research Electronic Data Capture; Vanderbilt University) surveys and an optional qualitative exit interview. Analyses will include hybrid inductive-deductive analyses for qualitative data and assessment of changes in our intervention targets and outcomes using *t* tests and correlation analyses.

**Results:**

In phase 1, caregivers reported interest in a brief, online stress management program. Participants held misconceptions about mindfulness and self-compassion, but after detailed explanation thoughts, these skills could be helpful when directly linked to implementation during caregiving routines. Phases 2 and 3 will be completed by the end of 2025.

**Conclusions:**

We describe the protocol for the Supporting Our Caregivers in ADRD Learning study, as well as the development and feasibility testing of the Mindful and Self-Compassionate Care intervention. Future work will include a fully powered efficacy-effectiveness randomized controlled trial.

**Trial Registration:**

ClinicalTrials NCT05847153; https://clinicaltrials.gov/study/NCT05847153; and ClinicalTrials.gov NCT06276023; https://clinicaltrials.gov/study/NCT06276023

**International Registered Report Identifier (IRRID):**

DERR1-10.2196/58356

## Introduction

More than 6 million people live with dementia in the United States and this number is projected to more than double by 2050 [1]. Characterized by a loss of cognitive functioning due to an irreversible loss of neurons (can be caused by many different diseases), dementia encompasses Alzheimer disease, dementia with Lewy bodies, vascular dementia, and frontotemporal dementia [2].

The cost of dementia impacts health care systems, individuals, and society in general. According to the Alzheimer’s Association, in 2024, the total costs of care will reach US $360 billion, with US $91 billion borne from out-of-pocket spending. These costs do not account for informal caregiving by relatives, friends, or neighbors who are unpaid for their services [3]. Indeed, in 2023, it is estimated that over 11 million unpaid caregivers provided over 18.4 billion hours of care for people living with Alzheimer disease and related dementias (ADRD) [3].

The extent of care provided by caregivers often results in a great deal of psychological, physical, emotional, and functional stress with 40% of caregivers citing a level of stress that interferes with their ability to care for themselves and their loved ones [1,4,5]. Despite their critical role in supporting people with ADRD, caregivers are often described as “hidden patients” whose health care needs often go underrecognized and undertreated [6]. A growing body of literature suggests that caregivers are at risk for decreased quality of life, increased depression as well as negative health outcomes [1,3]. Most caregivers lack effective nonpharmacological interventions to manage the stress associated with caregiving and their care recipient’s behavioral and mood symptoms. For instance, individuals living with ADRD may present with challenging behaviors such as aggression, agitation, and apathy [7,8]. Such behaviors have been associated with heightened emotional distress (eg, symptoms of depression and anxiety), decreased well-being, and increased risk for morbidity and mortality in both caregivers and care recipients [3,4].

Addressing a caregiver’s stress has the potential to mitigate risk for the exacerbation of the caregiver’s chronic health problems and to improve emotional and health outcomes for people living with ADRD [4,5]. Preliminary data [8] indicate ADRD caregivers desire real-time guidance and support to learn about emotional regulation, self-compassion, and behavioral management skills that can help them navigate stress related to their care-recipients’ challenging and developing needs.

Many of the current caregiver support programs do not fully meet the psychological and social needs of stressed caregivers for 3 main reasons. First, support groups may not teach evidence-based behavioral management skills that caregivers report they need to manage the challenging behaviors of people living with dementia successfully. Second, behavioral management skills interventions, while available, often do not teach emotional regulation skills, which are necessary to foster the caregiver’s ability to manage their care recipient’s behaviors. Third, though mindfulness and self-compassion interventions are theoretically based, effective solutions for managing stress and distress among caregivers have rarely been applied to managing common challenging behaviors experienced by care recipients [9,10].

To address the need for a feasible, acceptable, effective, and scalable stress management program to reduce the stress of ADRD caregivers, we created the Supporting Our Caregivers in ADRD Learning (SOCIAL) study which aimed to identify and manage stressful situations that arise for caregivers. We hypothesize that learning and practicing skills with mindfulness and self-compassion will decrease loneliness, increase the quality of relationships, and increase caregiver’s self-efficacy when managing challenging patient behavioral symptoms.

## Methods

### Conceptual Model

**Figure 1:**
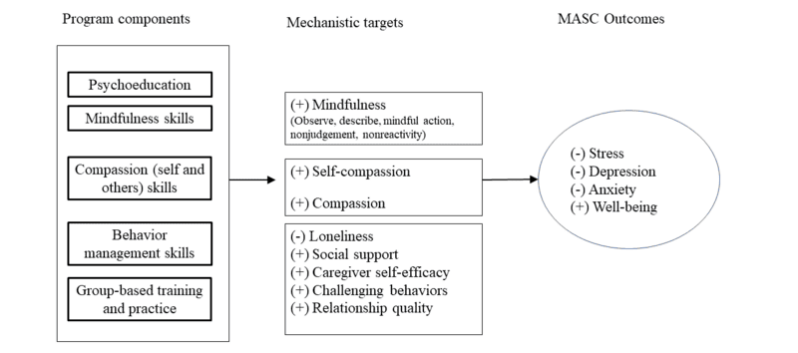
Mindful and Self-Compassionate Care program intervention model. MASC: Mindful and Self-Compassionate Care.

Our conceptual model (Figure 1) draws from principles and skills from Kabat Zinn’s Mindfulness-Based Stress Reduction program [11], Kristin Neff’s Mindful Self-Compassion program [12], and Laura Gitlin’s caregiver guide to managing challenging behaviors [13]. Our multicomponent program incorporates (1) Mindfulness-Based Stress Reduction program’s mindfulness skills (observe, describe, mindful action, nonjudgement, and nonreactivity) to support emotional regulation during challenging dementia behaviors; (2) Christine Neff’s compassion and self-compassion skills (self-kindness and common humanity) to encourage caregivers to be kind to themselves and encourage connection with their care recipients and others; and (3) Laura Gitlin’s behavioral management strategies to navigate patient behavioral symptoms. Psychoeducation and group-based practice will provide an opportunity to learn how to integrate skills into the daily caregiver role. The Mindful and Self-Compassionate Care (MASC) program’s mechanistic targets are hypothesized to act synergistically, leading to reduced stress, symptoms of depression, and anxiety, and improved well-being (Figure 1).

This paper describes preliminary qualitative input from 28 caregivers in 5 focus group interviews (phase 1) used to guide the MASC intervention’s development. We describe the design of the open pilot (phase 2) and the feasibility randomized controlled trial (RCT; phase 3). Ultimately, we aim to provide a framework for increasing access to evidence-based psychoeducation interventions for caregivers of people with ADRD.

### Study Design

**Figure 2:**
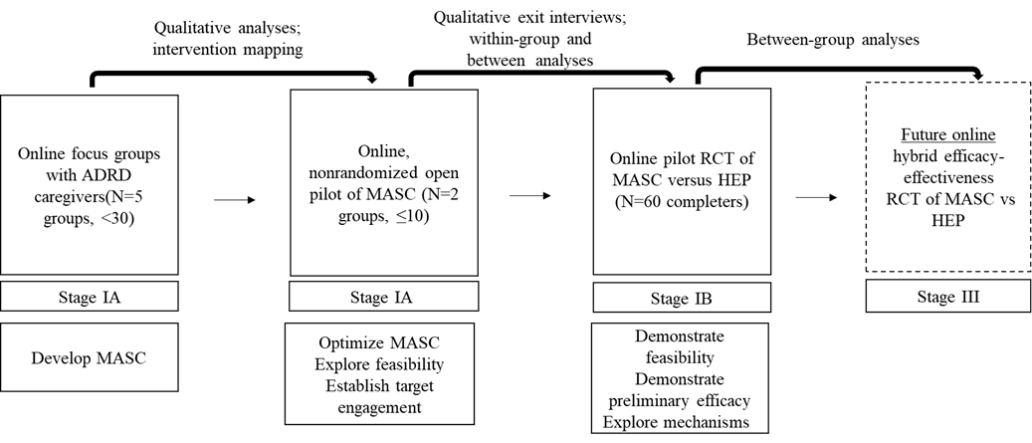
Mindful and Self-Compassionate Care Program intervention development, optimization, and testing following the National Institute of Health stage model and Science of Behavior Change principles [14]. ADRD: Alzheimer’s Disease and Related Dementias; HEP: Health Education Program; MASC: Mindful and Self-Compassionate Care; RCT: randomized controlled trial.

This study consists of 3 phases, that are a qualitative phase, an open pilot phase, and a feasibility RCT (Figure 2 [14]).

### Ethical Considerations

The study has been reviewed and approved (phase 1 protocol 2022P002037, phase 2 protocol 2023P001130, and phase 3 protocol 2023P003628) by the Massachusetts General Brigham institutional review board. Eligible participants will engage in an informed consent discussion with the research assistant surrounding study procedures, expectations, potential risks and benefits of participation, and contact information for further questions. They will also be informed that participation at every stage is voluntary, and they can withdraw from the study at any time. To protect against privacy risks, all participants will be assigned a unique study ID to keep their identity confidential. In addition, all data will be collected and stored within the Massachusetts General Brigham secured REDCap (Research Electronic Data Capture; Vanderbilt University). Compensation varies depending on the stage of the study; up to US $50 (phase 1), up to US $60 (phase 2), and up to US $120 (phase 3).

### Setting and Participants

Participants for all phases of the study will be English-speaking adults (aged 18 years or older) who identified as informal caregivers to individuals with ADRD. Participants must live with and have provided more than 4 hours of direct unpaid care per day in the past 6 months. In addition, caregivers must endorse stress (Perceived Stress Scale [PSS]–4 [15] score ≥6) and self-report that they managed one or more behavioral symptoms in the past month as defined by the Neuropsychiatric Inventory Questionnaire [16]. Caregivers were excluded if the care recipient had a recent change in psychiatric medications, had used a mindfulness app or any meditation for more than 60 minutes per week in the past 6 months, or were participating in another clinical trial for caregivers. All focus groups and interventions were conducted through a secure online platform.

### Recruitment, Screening, and Enrollment

Caregivers were recruited from local sources, including the Dementia Care Collaborative program at Massachusetts General Hospital, the Massachusetts Alzheimer’s Disease Research Center, the Massachusetts General Brigham research posting site, nationally, through the National Alliance of Caregiving and ADRD caregiver-specific social media pages (ie, Facebook [Meta], Instagram [Meta], and Twitter [rebranded as X]). All recruitment efforts were documented in REDCap (version 14.0.27) to solidify the recruitment plan for subsequent phases of the study.

### Phase 1: Focus Groups

A total of five 60-minute qualitative focus groups were conducted with caregivers through secure live videoconferencing to elicit feedback on proposed content, skills, and logistics (length and duration of sessions, online vs in person). Participants responded to prompts surrounding caregiver needs, proposed program content, and structure as well as barriers and facilitators to participation. Focus groups included modeling of mindfulness and self-compassion skills as well as discussions on a case-based scenario to gauge interest in and reactions to tailoring the program content. Focus groups were recorded and transcribed [17]. Participants were also given the opportunity to complete an optional exit survey comprised of the outcome measurement tools being used in phases 2 and 3. In total, 24 caregivers completed the exit survey and 21 caregivers provided additional written feedbacks.

### Data Coding and Analysis

Rapid data analysis (RDA) guided immediate refinement of the intervention, based on the Framework Method [18,19]. Analysis comprised of 3 researchers (SMS, AT, and XXX) coding data into superordinate themes and subthemes using hybrid deductive-inductive analysis [20]. Our RDA template was organized into certain domains, such as population-specific caregiver experiences, program content, and barriers and facilitators for program implementation. The completed RDA template was reviewed by the study team and entered into a matrix of responses which informed manual and procedural adaptations before the open pilot. The matrix was organized in a hybrid inductive-deductive manner based on a combination of the domains from the RDA template and the information that emerged from qualitative focus groups that was most useful in guiding program adaptations.

Formal qualitative analyses and in-depth synthesis of the qualitative data are forthcoming. Thematic analyses will be supported by NVivo 12 (Lumivero). A codebook will be developed using a hybrid deductive-inductive approach wherein codes are created based on a priori categorizations using the Framework method [21]. A second revision will create additional codes based on novel content identified directly from the data and through the analysis process. Furthermore, 2 trained research assistants will code the transcripts under the guidance of the investigative team. Discrepancies between coders will be discussed until a consensus is reached.

### Phase 2 and 3: Open Pilot of Mindful and Self-Compassionate Care (Phase 2) and RCT of SOCIAL

#### Phase 2 and 3 Study Design

The open pilot (phase 2) of MASC will be comprised of a 6-session open pilot delivered through live video to ADRD caregivers who meet eligibility criteria (Figure 1). MASC will be delivered by a trained clinical social worker to 2 groups of 6-10 caregivers. Caregivers complete a questionnaire packet focused on mental well-being as well as questions about their experiences as caregivers for someone living with dementia. Participants will participate in a weekly, 60-minute, online group training session for 6 weeks followed by an exit interview. The open pilot will be used to refine the intervention and the manual used in the phase 3 feasibility RCT of MASC versus a Health Education Program (HEP; the SOCIAL Study).

#### Setting and Participants of Phases 2 and 3: Recruitment, Screening, and Enrollment

Initial recruitment, screening, and enrollment procedures will be identical to those described for the focus groups. Potential participants recruited nationally will indicate their interest by completing the REDCap screener using the QR code on the flyer or by emailing the research assistants. Research assistants will reach out to eligible caregivers and engage in a discussion that includes an explanation of study procedures, potential risks and benefits of participation, and contact information. All eligible and available caregivers will be emailed a link to the baseline questionnaire in REDCap and offered assistance with questionnaire completion. Caregivers who provide consent but are unavailable to participate in the next available group will be placed on a waitlist for the next group cohort. Assigned participants will have the opportunity to attend a Zoom practice session with the research assistant to problem-solve any technical challenges.

#### Phase 2 and 3 Mindful and Self-Compassionate Care Intervention Content

Participants receive the 6-week MASC intervention including the manual, web resources, and live intervention. Session content is detailed in Table 1. Broadly, each session will include psychoeducation on each program skill, skill practice, strategies to incorporate the skill into the caregiver experience, and strategies for sustained practice. Participants will attend the session using a tablet or computer and will be encouraged to use the video feature to interact “face-to-face” during each video session. Participants will complete weekly “homework” consisting of skills practice audio exercises that allow them to continue to engage with the skills that they are learning outside of the sessions.

**Table 1:** Mindful and Self-Compassionate Care program content.

Session	MASC^a^ topic	Content and skills
1	Getting to know your stress response	Psychoeducation on stress response and sources of stress. Overview of program goals, skills and conceptual model, and goal settings.
2	Introduction to mindfulness	Benefits of mindfulness and meditation for managing caregiver stress. Incorporation of mindfulness into caregiving tasks and daily life.
3	The skill of compassion and managing challenging behaviors	Identifying sources of support and self-care. Incorporation of compassion into caregiving tasks and daily life. Introduction to challenging behaviors.
4	The skill of self-compassion while caregiving	Integrating mindfulness and self-compassion with behavioral management skills.
5	Communication skills	Reframing challenging behaviors as a form of communication as the brain degenerates. Anticipating and problem-solving communication and behavior challenges.
6	Putting it all together	Program review and strategies for continued practice (skills practice; continuing to work on individual goals).

^a^MASC: Mindful and Self-Compassionate Care.

#### Phase 2 and 3 Treatment Fidelity

Adherence checklists will ensure all components of the intervention are delivered in compliance with the study protocol. Clinicians write session summaries to capture session content and any issues that arise regarding individual concerns and progress. Sessions will be recorded, and independent reviewers will review 20% of the sessions to assess for fidelity [22]. Feedback will be provided in weekly supervision, led by one of the study’s principal investigators (A-MV). Participants who miss a session will be offered one-on-one make-up sessions. Home practice will be tracked through daily, 1-question yes or no surveys asking about whether they have engaged in the home practice that day sent by REDCap emails. These procedures follow the NIH (National Institutes of Health) Science of Behavior Change recommendations and have been used by the multiple principal investigators of this study in prior clinical trials [14].

#### Phase 2 and 3 Primary Outcomes

The primary outcomes for the open pilot (phase 2) and the RCT (phase 3) include feasibility of recruitment, assessments, and quantitative measures, credibility, accessibility, and fidelity (Table 2). Our secondary outcomes (Table 3) will explore indicators of the preliminary efficacy of the same quantitative measures. The feasibility of recruitment and retention will be assessed using proportions. Satisfaction and credibility will be assessed with proportions of scores over the midpoint of the Client Satisfaction and Credibility and Expectancy questionnaires [23,24].

**Table 2:** Phase 2 and 3 primary outcome benchmark definitions.

Measure	Brief description
Feasibility of recruitment	Assessed by the proportion of caregivers who were eligible to enroll, proportion of caregivers recruited from each recruitment source, and the proportion of racial and ethnic minorities recruited and enrolled across the entire sample.
Feasibility of randomization	Assessed by the proportion of randomized caregivers that completed the posttest.
Feasibility of assessment measures	Assessed by the proportion of participants with less than 25% of their questionnaires missing.
Adherence to treatment	Assessed by the proportion of randomized caregivers that attended at least 4 of the 6 sessions for both the intervention (MASC^a^) and control group (HEP^b^).
Therapist fidelity	Assessed by therapist’s ability to deliver the content of each session (through therapist-completed adherence checklists) and therapist fidelity (through cotherapist’s review of recorded sessions).
Credibility and expectancy	Assessed with the Credibility and Expectancy Questionnaire (CEQ) [23] which captures participants’ perceptions on whether treatment will work after learning about the study.
Satisfaction with the program	Assessed by the proportion of participants that score above the Client Satisfaction Questionnaire (CSQ-3) [24] midpoint which assesses caregivers’ satisfactions with the intervention.
Patients’ perception of improvement	Assessed by the proportion of caregivers that report improvement in stress, depression, anxiety, and well-being [25].
Adherence to home practice	Assessed by the proportion of caregivers that have completed more than 50% daily home practice exercises (assessed by REDCap^c^).

^a^MASC: Mindful and Self-Compassionate Care.

^b^HEP: Health Education Program.

^c^REDCap: Research Electronic Data Capture.

**Table 3:** Phase 2 and 3 secondary outcome measures.

Measure	Administration time frame	Brief description
Symptoms of depression	Baseline, postintervention, and follow-up	The Center for Epidemiological Studies-Depression Scale (CES-D) [26] is a 20-item scale widely used with ADRD^a^ caregivers to assess depression.
Mindfulness	Baseline, postintervention, and follow-up	The Applied Mindfulness Process Scale (AMPS) [27] is a 15-item scale that assesses caregivers’ ability to apply mindfulness to everyday challenges.
Perceived stress	Baseline, postintervention, and follow-up	The Perceived Stress Scale (PSS-10) [28] assesses perceived stress using a 5-point Likert scale.
Symptoms of anxiety	Baseline, postintervention, and follow-up	The State Trait Anxiety Inventory (STAI) [29] form Y (20 items) assesses anxiety symptoms in response to stressful situations. State Trait Anxiety Inventory has been successfully used with ADRD caregivers.
Self-compassion	Baseline, postintervention, and follow-up	The Self-Compassion Scale-Short Form (SCS-SF) [30] has 12 items that assess self-kindness.
Compassion	Baseline, postintervention, and follow-up	The Compassion Scale (CS) [31] has 16 items assessing common humanity, kindness toward others and ability to understand the suffering or challenges of others.
Distress due to challenging behaviors	Baseline, postintervention, and follow-up	The Neuropsychiatric Inventory Caregiver Distress Scale (NPI-Q) [16] has 12 items assessing distress associated with behaviors of patient’s suffering from dementia.
Caregiver self-efficacy	Baseline, postintervention, and follow-up	The Revised Scale for Caregiver Self-Efficacy (RSCSE) [32] (8 items) assesses domains of self-efficacy including obtaining respite, responding to disruptive patient behaviors, and controlling upsetting thoughts.
Well-being	Baseline, postintervention, and follow-up	The World Health Organization-Five Well-Being Index (WHO-5) [33] has 5 items assessing emotional well-being.
Loneliness	Baseline, postintervention, and follow-up	The University of California Los Angeles 3-item loneliness scale [34] assesses relational connectedness, social connectedness, and self-perceived isolation.
Social support	Baseline, postintervention, and follow-up	The Interpersonal Support Evaluation List short form (ISEL) [35] has 12 items assessing appraisal, belonging, and tangible social support.
Relationship quality	Baseline, postintervention, and follow-up	The Dyadic Relationship Scale (DRS) [36] has 11 items that assess negative and positive interactions between caregivers and their care recipient.

^a^ADRD: Alzheimer disease and related dementias.

Secondary outcomes include stress [28], depression [26], anxiety [29], and well-being [33] through the mechanistic targets of mindfulness [27], caregiver self-efficacy [32], social support [35], loneliness [34], compassion for self and others [30,31], behavioral symptom management [16], and relationship quality [36]. Participants will complete assessments within 1 week of starting the first session and will provide feedback at the end of session 6 and at 3 months. Study assessments are presented in Table 3. The self-reported measures and assessment domains align with the purpose of the study and with recommendations for feasibility trials. The measures are reliable and valid for dementia caregivers [37,38].

#### Phase 2 Data Analysis

The feasibility of recruitment and retention will be assessed using proportions defined in Table 2. Satisfaction and credibility will be assessed with proportions of scores over the midpoint of the Client Satisfaction [24] and Credibility and Expectancy [23] questionnaires. Qualitative data from exit interviews will be analyzed using procedures outlined in phase 1. Given the exploratory nature of this open pilot, we do not anticipate statistical significance [39]. Pre- and postchanges in intervention targets and outcomes will be analyzed using *t* tests and explore correlations between change scores in targets and outcomes in the pre- and post–follow-up surveys.

All outcomes will report 95% CIs consistent with recommendations for pilot trials [40]. Procedures, content, and measures will be revised based on quantitative and qualitative data.

#### Phase 2 Open Pilot Intervention Refinement

The MASC intervention will be refined based on overall findings of feasibility, acceptability, credibility, fidelity, preliminary efficacy outcomes, and 30-minute exit interviews from study participants.

#### Phase 3 Health Education Program

Phase 3 SOCIAL study will include a 6-session HEP comparator which will mimic the dose, format, and duration of MASC. The topics of each session will contain dementia-related educational information adapted from the National Alliance of Caregiving. HEP will include topics of (1) the stress of caregiving, (2) sleep hygiene, (3) physical activity as a dementia caregiver, (4) nutrition, (5) developing healthy eating habits, and (6) a review of program content and postprogram plans. HEP will not teach any of the mindfulness, self-compassion, and behavioral management skills that are hypothesized to be responsible for improvements in the mechanistic targets and through them, improvements in outcomes.

#### Phase 3 Randomization Strategy

A 2:1 MASC:HEP randomization will be prepared by an unmasked statistician using REDCap to create permuted blocks stratified by gender. To maintain blinding and masking, the programs will be labeled as SOCIAL 1 (MASC) and SOCIAL 2 (HEP). Both MASC and HEP groups will be run by an experienced clinical social worker trained in the intervention and the HEP and will work from updated facilitator manuals. The same social worker will run the intervention and comparison groups to control for nonspecific factors.

#### Phase 3 Analysis

The RCT will follow the same general analysis plan as the phase 2 open pilot with expansion to include the feasibility of randomization. Required benchmarks are depicted in Table 4. We will report benchmarks for both MASC and the HEP, consistent with recommendations for stage 1B trials [14]. We estimate between-group differences in change from baseline in intervention targets and outcomes to posttest and 3 months as proof of concept. Variance components and effect sizes will be estimated for each outcome [41]. The study will not be powered for stringent inferential testing of efficacy unless an overwhelming benefit is observed from the MASC program. The variance estimates from repeated-measures analysis of data on PSS-10 scores in tandem with the minimal clinically significant difference will guide the design and power calculations for the future hybrid efficacy-effectiveness trial [42]. We will compute the effect sizes of changes in these scores along with 95% CIs. Analyses will be repeated for all targets and outcomes. For example, proofs of concept or preliminary efficacy will be demonstrated if decrease in stress (PSS-10) in the intervention group from baseline to posttest is greater than in the educational control and the 95% CI includes the minimal clinically significant difference. We chose this benchmark because PSS-10 will be the primary outcome in the future R01. If the benchmark is not met, revisions will be required before conducting a fully powered RCT. The correlation of changes will be assessed in the mechanistic targets (mindfulness, caregiver self-efficacy, social support, loneliness, compassion, self-compassion, behavioral symptom management, and relationship quality) with changes in the outcomes (stress, depression, anxiety, and well-being). We hypothesize that the intervention (targeting mindfulness, self-efficacy, coping, social support, and interpersonal bonds) is a mechanism for effecting change in outcomes. Outcomes will be assessed post intervention and at 3 months.

**Table 4:** Phase 3 outcome benchmarks.

Measure	Brief description
Feasibility of recruitment	≥70% of caregivers who are eligible will enroll; ≥38% of caregivers are from racial and ethnic minorities.
Feasibility of randomization	≥70% of caregivers randomized will complete the posttest.
Feasibility of assessment measures	≥70% of caregivers will have less than 25% missing questionnaires.
Adherence to treatment	≥70% of randomized caregivers will attend at least 4 (out of 6) sessions for both MASC^a^ and HEP^b^.
Therapist fidelity	≥75% fidelity (checklists and audio recordings) for ≥70% caregivers.
Credibility and Expectancy Score	≥70% of caregivers with score over the scale midpoint.
Client Satisfaction Score	≥70% of caregivers with score over the scale midpoint.
Patients’ perceptions of improvement	≥70% of caregivers report improvement in stress, depression, anxiety, and well-being.
Adherence to home practice	≥70% of caregivers will complete more than 50% daily home practice exercises (assessed through REDCap^c^).

^a^MASC: Mindful and Self-Compassionate Care.

^b^HEP: Health Education Program.

^c^REDCap: Research Electronic Data Capture.

## Results

### Caregiver Experiences

Phase 1 is complete. Findings from our rapid data analysis include confirmation from care providers that caregiving was rewarding but also difficult. Caregivers spoke of difficulties associated with the emotional toll, that were linked to (1) their emotions (sadness and grief) prompted by seeing their care recipient decline mentally and physically or (2) the impact of the difficulty in managing the rapidly changing emotions or moods of their care recipients. Caregivers affirmed the challenges of caregiving for a person with ADRD, including navigating behavioral symptoms and their impact on daily activities, such as bathing, taking medications, and going to doctors’ appointments. Caregivers primarily cited using music and touch as ways they addressed challenging behaviors and expressed that they would like to learn more skills to use in the moment.

### Feedback on Skills

Many caregivers were unfamiliar with the concept of mindfulness and had not used it before. After being introduced to the concept of mindfulness and its benefits, they felt that mindfulness would help them manage caregiving stress. After being guided through a short mindfulness exercise they reported that this skill would be feasible to learn and implement. When caregivers were presented with the concept of self-compassion, many had misconceptions about it and its applicability to caregiving. When provided with information about the meaning of self-compassion, they were unsure if it was possible to be both self-compassionate and tend to their duties as a caregiver—but noted a willingness to try. Caregivers expressed interest in learning practical strategies to manage the neuropsychiatric symptoms of their care recipient and affirmed the rationale for using mindfulness and self-compassion to regulate their emotions.

### Delivery and Format

Participants provided feedback on the delivery and format of the group offering suggestions to support their learning and engagement in the program content. Caregivers suggested that audio recordings would be beneficial for ensuring the active practice of the skills independently. They had mixed feelings about the assignment of homework to reinforce skills. The caregivers overwhelmingly expressed a desire for a 1-hour online format.

## Discussion

### Anticipated Findings

This paper describes the process of developing, optimizing, and testing a clinical intervention for stressed caregivers through psychoeducation and skills training in mindfulness, self-compassion, and management of behavioral symptoms of dementia. The aim of this intervention is to fill the gap in nonpharmacological interventions to meet the psychological and practical needs of stressed caregivers of persons with ADRD with challenging behaviors and personalities.

Findings from the rapid data analysis guided the development of the intervention and the group format to meet the needs expressed by dementia caregivers and prevent factors that could hinder caregivers’ sustained participations. For example, MASC’s development incorporated facilitators to caregiver’s participation through the addition of an online delivery of program content, length, and duration with 6 sessions lasting for 60 minutes. Feedback on the program format guide indicated the need to offer the program online at flexible times that work best for most caregivers.

To date, most caregiver support groups are either unstructured or focused on strategies to address communication or behavioral symptom strategies [5]. Lack of attention to the psychological needs of the caregiver in many of these interventions may contribute to their modest ability to reduce caregiver stress and behavioral symptoms. Fostering self-regulation skills and strategies as outlined in the MASC intervention may augment caregivers’ abilities to navigate the array of challenges faced in caring for someone with dementia and reduce their stress at the same time. Given the difficulties grasping mindfulness and self-compassion concepts, MASC directly addresses these barriers by delivering simple lay language and easy to engage exercises linked with ADRD caregiving challenges. Content specifically addresses common challenging behaviors and associated stressors for both the caregiver and the care recipient.

### Conclusion

MASC’s skills grounded in mindfulness, self-compassion, and behavioral management aim to address stress, depression, loneliness, and anxiety. Similarly, content was developed to improve well-being through caregiver self-efficacy, social support, mindfulness, self-compassion, compassion, and improved relationship quality. We hypothesize that the combination of evidence-based mindfulness and self-compassion skills with behavioral management skills within a multi-component program will increase intervention potency and efficiently support caregivers of people with ADRD. By reducing stress in caregivers of people with ADRD and evaluating this intervention with a rigorously designed control condition, the proposed work has the potential to improve caregivers’ well-being and elucidate the mechanisms most relevant for improving caregiver outcomes.

## Appendix

Multimedia Appendix 1 Peer-review report by the National Institute on Aging (NIA) Special Emphasis Panel - Dementia Care (National Institutes of Health, USA).

## Abbreviations

ADRD: Alzheimer disease and related dementias
HEP: Health Education Program
NIH: National Institute of Health
MASC: Mindful and Self-Compassionate Care
PSS: Perceived Stress Scale
RCT: randomized controlled trial
RDA: rapid data analysis
REDCap: Research Electronic Data Capture
SOCIAL: Supporting Our Caregivers in ADRD Learning

## Data Availability

The data sets generated during and/or analyzed during this study are not publicly available until they have been deposited at the National Archive of Computerized Data on Aging, located within the Inter-university Consortium for Political and Social Research, and funded by the National Institute on Aging, but are available from the corresponding author on reasonable request.
