# Supplementary material for: Intervention for the Management of Neuropsychiatric Symptoms to Reduce Caregiver Stress: Protocol for the Mindful and Self-Compassion Care Intervention for Caregivers of Persons Living With Dementia
Source: JMIR Res Protoc. 2024 Oct 11;13:e58356. doi: 10.2196/58356 (PMC11512127; doi:10.2196/58356)
Supplement: Multimedia Appendix 1 [file resprot_v13i1e58356_app1.pdf]

SUMMARY STATEMENT
(Privileged Communication)

PROGRAM CONTACT:
LISA ONKEN
(30I) 496-3131
lonken@nia.nih.gov

Release Date: 04/05/2022
Revised Date:

Application Number: 1 R01 AG078204-01

Principal Investigators (Listed Alphabetically):

RITCHIE, CHRISTINE S (Contact)
VRANCEANU, ANA-MARIA

Applicant Organization: MASSACHUSETTS GENERAL HOSPITAL

Review Group: ZAG1 ZIJ-D (M2)
National Institute on Aging Special Emphasis Panel
Dementia Care

Meeting Date: 03/17/2022
Council: MAY 2022
Requested Start: 07/01/2022
RFA/PA: PAR21-307
PCC: 2BPDILO

Project Title: The Mindful and Self-Compassionate Care Program (MASC): Reducing Stress for Caregivers of Persons with Dementia
SRG Action: Impact Score:33
Next Steps: Visit https://grants.nih.gov/grants/next_steps.htm
Human Subjects: 30-Human subjects involved - Certified, no SRG concerns
Animal Subjects: 10-No live vertebrate animals involved for competing appl.
Gender: 1A-Both genders, scientifically acceptable
Minority: 1A-Minorities and non-minorities, scientifically acceptable
Age: 3A-No children included, scientifically acceptable

ADMINISTRATIVE BUDGET NOTE: The budget shown is the requested budget and has not been adjusted to reflect any recommendations made by reviewers. If an award is planned, the costs will be calculated by Institute grants management staff based on the recommendations outlined below in the COMMITTEE BUDGET RECOMMENDATIONS section.

RESUME AND SUMMARY OF DISCUSSION: This new application, for Dementia Care and Caregiver Support Intervention Research (R01, Clinical Trial Required) is tendered by Massachusetts General Hospital on behalf of Principal Investigator (PI) Dr. Kristine Ritchie and Multiple Principal Investigator (MPI) Dr. Ana-Maria Vranceanu. An MPI Leadership Plan is included. It requests three years of funding to develop, optimize, and establish proof of concept for the Mindfulness, Self-Compassionate Care (MASC) Program for stressed caregivers of persons with Alzheimer's disease and related dementias (ADRD). To accomplish this, the research team will use focus groups to develop the MASC; optimize the MSAC and conduct an open pilot to explore feasibility, benchmarks, target enrollment, and gauge potential improvement in stress, depression, anxiety, and well-being; and conduct a pilot randomized clinical trial of MASC versus a Health Education Program control to establish feasibility benchmarks and preliminary efficacy, and explore potential improvement via mechanistic targets. If successful, this work has the potential to offer scalability of MASC and improved care for ADRD caregivers across the US. This work builds on Dr. Ritchie's experience in research on geriatrics and palliative care aimed to optimize quality of life for those with chronic conditions and multimorbidity, including persons living with dementia and their caregivers. Dr. Vranceanu has experience in mind-body intervention development and optimization and virtual or fully remote mind-body clinical trials. Thus, Drs. Ritchie and Vranceanu are well-suited to lead this research effort. Other notable strengths include the strong interdisciplinary team (and their experience with the NIH Stage Model), history of collaboration between the two PIs, well-conceived and justified aims, rigorous approach, well-developed benchmarks, national recruitment and recruitment of diverse samples, discussion of practical considerations (e.g., engaging people), well-considered conceptual model, companion web tool, clearly defined outcomes, and strong planned analysis. However, several weaknesses temper enthusiasm, including the arbitrary distinction between primary and secondary outcomes, the reliance of assessments on self-report, lack of clarity regarding what constitutes "real world" in the population being studied, exclusion of Spanish speakers, lack of clarity about mechanisms, and lack of demonstration of efficacy of intervention on caregiver stress. There was disagreement about whether achievement of aims is contingent on success of prior aims, and also about whether the intervention could be implemented successfully in real-world settings.

DESCRIPTION (provided by applicant): Within a 3-year R01 we will use the NIH stage model and principles from the Science of Behavioral Change to develop, optimize and establish proof of concept for the Mindful, Self-Compassionate Care Program (MASC) for stressed caregivers of persons with ADRD with challenging behaviors. In aim 1, we will use qualitative focus groups to develop MASC (NIH stage 1A; up to 30 caregivers; n=4 groups). In aim 2, we will optimize MASC and conduct an open pilot with exit interviews to explore feasibility benchmarks, target engagement and signal of improvement in stress, depression, anxiety and well- being (NIH stage 1A; up to 10 caregivers; n=1 group). In aim 3, we will conduct a pilot RCT of MASC versus a Health Education control to establish feasibility benchmarks and preliminary efficacy and explore evidence for potential mechanisms of improvement through the proposed mechanistic targets (N=80/c0 completers). MASC combines evidence- based skills from mindfulness programs, self-compassion programs, and behavioral management programs. MASC structure and components are designed to facilitate uptake, skill practice, and sustainability of improvements. MASC has 6 sessions and is delivered virtually in groups. MASC has an associated web platform that facilitates skills practice and is available to caregivers after the group ends. Our guiding hypothesis is that MASC skills of mindfulness, self-compassion, and behavioral management will interact and lead to decreased stress, depression and anxiety and improved well-being, through decreased loneliness, increased caregiver self-efficacy, social support, mindfulness, self-compassion, compassion, and improved relationship quality. Our team is well positioned to conduct this study with specific experience in mindfulness and self-compassion programs; intervention development, feasibility testing and clinical trials; ADRD caregivers; recruitment of national, geographically diverse samples. At the end of the 3-year period of this R01 we will have clear evidence of the feasibility of study procedures, randomization, acceptability, credibility, adherence, ability to retain and recruit as well as its proof of concepts in reducing stress in caregivers of people with ADRD and proposed mechanisms. Results from this R01 study will be used to apply for funding to conduct a fully powered hybrid efficacy-

effectiveness RCT. The goal is dissemination and implementation of MASC to reduce stress, among all in need caregivers of persons with ADRD.

PUBLIC HEALTH RELEVANCE: By reducing stress in caregivers of persons with ADRD, the proposed work has the potential to drastically improve emotional well-being and physical health in not only caregivers, but also the people they care for. MASC teaches meditation, self-compassion and behavioral management skills without the burden associated with traditional in person or virtual meditation-based interventions. As such, there is tremendous opportunity for scalability and improved care for ADRD caregivers across USA.

DISCLAIMER: Please note that the following critiques were prepared by the reviewers prior to the Study Section meeting and are provided in an essentially unedited form. While there is opportunity for the reviewers to update or revise their written evaluation, based upon the group’s discussion, there is no guarantee that individual critiques have been updated subsequent to the discussion at the meeting. Therefore, the critiques may not fully reflect the final opinions of the individual reviewers at the close of group discussion or the final majority opinion of the group. Thus, the Resume and Summary of Discussion is the final word on what the reviewers actually considered critical at the meeting.

CRITIQUE 1:

Significance: 1
Investigator(s): 1
Innovation: 1
Approach: 2
Environment: 1

Overall Impact:
This is outstanding application from an excellent group of investigators. The planned innovation and approach are excellent. The aims are well-conceived, and very systematic and rigorous. This application is impressively prepared and is a pleasure to read.

1. Significance:
Strengths
- The significance of this application is high.
- The application is highly responsive to the funding opportunity.
- When caregivers lack the skills necessary to manage behavioral symptoms associated with Alzheimer’s disease and related dementia (ADRD), they are more likely to report greater stress, symptoms of depression and anxiety, and poor self-rated health.

Weaknesses
- None identified.

2. Investigator(s):
Strengths
- The applicant team is well-qualified to conduct the proposed work. The interdisciplinary team, collectively, has expertise in mindfulness and self-compassion programs, intervention development, feasibility testing and clinical trials, ADRD caregivers, and recruitment of national, geographically diverse samples.
- They additionally have experience using the NIH model to develop and optimize interventions, and in recruiting and retaining ADRD caregivers, and racial and ethnic minorities.
- The two Principal Investigators (PIs), Drs. Christine Ritchie and Ana-Maria Vranceanu, have a history of collaboration.

Weaknesses
- None identified.

3. Innovation:
Strengths
- The application states that this would be the first intervention for caregivers that incorporates emotional regulation, self-compassion, and behavioral management skills into the therapeutic model to help individuals successfully navigate the stress of the caregiving experience.
- This adds to the growing (and important) literature on interventions for caregivers.

Weaknesses
- None noted.

4. Approach:
Strengths
- An intervention that combines a focus on evidence-based mindfulness and self-compassion skills with behavioral management skills appears very promising for reducing stress among caregivers.
- The research team has carefully considered a wide array of practical considerations relevant to participant engagement in the intervention (e.g., virtual delivery, manageable length of sessions, use of lay language).
- The focus on present moment awareness and empathy toward self and others as mechanisms to improve stress is compelling.
- The conceptual model presented in the application is also compelling and well-considered.
- The use of companion web tool with videos, exercises, etc., to reinforce the therapeutic model, is a strength. This may be one of the most important components of the model – being accessible anytime (increasingly scalable potential), and delivered with fidelity.
- Planned national recruitment is excellent.
- Planned recruitment that includes a focus on diversity and inclusion is a strength.
- Planned aims, aligned with the NIH Stage Model of Intervention Development, are well-conceived and systematically build on themselves.
- Fidelity procedures are important.
- Attention control group is appropriate.
- Criteria for achieving targeted outcomes under Aim 3 activities (e.g., feasibility, preliminary efficacy) are very clearly defined.
- The planned analyses around mechanistic targets are excellent.
- Overall design considerations are well-justified.

Weaknesses
- Information is somewhat lacking regarding how the outcomes from Aim 1 activities will inform the intervention used in Aim 2, and how the outcomes from Aim 2 activities will inform the intervention used in Aim 3. There is very little detail on this.

5. Environment:
Strengths
- Excellent.

Weaknesses
- None identified.

Study Timeline:
Strengths
- Acceptable. The detailed milestones are well considered.

Weaknesses
- None identified.

Protections for Human Subjects:
Acceptable Risks and/or Adequate Protections.

Data and Safety Monitoring Plan (Applicable for Clinical Trials Only):
Acceptable.

Inclusion Plans:
- Sex/Gender: Distribution justified scientifically.
- Race/Ethnicity: Distribution justified scientifically.
- Inclusion/Exclusion Based on Age: Distribution justified scientifically.

Resource Sharing Plans:
Acceptable.

Budget and Period of Support:
Recommend as Requested.

CRITIQUE 2:

Significance: 5
Investigator(s): 2
Innovation: 5
Approach: 6
Environment: 1

Overall Impact:
The applicants draw from the NIH Stage Model and NIH-supported Science of Behavior Change to develop, optimize, and test a six-session behavioral intervention for caregivers of persons with dementia. The intervention is delivered virtually in groups. The primary outcome is decreased perceived stress. Several secondary outcomes and intervention targets will also be assessed. By the end of the proposed three-year project, the applicants believe they will have established the feasibility of study procedures and preliminary evidence of efficacy, including the hypothesized mechanism of action. Strengths of the application include the PIs, the environment at Massachusetts General Hospital (MGH), the delivery of the intervention via the web, and the plan to recruit a diverse sample. These strengths are outweighed by numerous conceptual and methodological weaknesses, including an incomplete analysis of barriers to longer term implementation; lack of clarity about the distinctions between primary outcomes, secondary outcomes, and intervention targets; an incomplete review and analysis of prior mindfulness interventions; an approach to the measurement of putative targets that is based entirely on self-report, conflates state and trait components, and ignores behavioral assessments or reports of skill use; and the exclusion of Spanish speakers. The proposed intervention is thus unlikely to have a significant, sustained scientific impact.

1. Significance:
Strengths
- Non-pharmacological interventions are needed for individuals who provide informal care for persons with ADRD.
- A web-based intervention has significant potential to enhance access to interventions that could mitigate, if not prevent, caregiving-related morbidity.
- Clinical trials that adopt an experimental therapeutics approach could isolate mechanisms of action.

Weaknesses
- The applicants wish to develop an intervention that is scalable and accessible, but they did not ground this project in foundational knowledge of barriers to the implementation of evidence-based behavioral interventions in community or clinical settings. This was not specifically required by the funding opportunity announcement (FOA), but it is a major weakness because the FOA does call for “activities that can lay the groundwork for principle-based ADRD care and caregiving interventions that can be delivered with fidelity in the real world.”
- The applicants do not specify the “real world” context (population, care delivery setting, payment mechanisms) in which this intervention has been developed. It is implicitly assumed that participants seeking care in an MGH clinic and those recruited over the internet inhabit the same “real world.” This does not appear like a viable premise and raising questions about the promise of scalability.
- Although the applicant team identified several practical issues relevant to participant engagement in the intervention (e.g., virtual delivery), they did not consider barriers at other levels of analysis (e.g., health-system, community). Failure to carefully analyze dissemination barriers in early research stages has undermined the dissemination of NIH-funded interventions for decades.
- The applicants did not expose the logic underlying basic design decisions. For example, it is not clear why the applicants classified stress as a primary outcome and designated other variables secondary outcomes or intervention targets. These designations appear arbitrary, undermining rigor.
- The applicants ignore literature on the adverse effects of mindfulness in some contexts. The assumption that mindfulness training would benefit participants is not well-justified.

2. Investigator(s):
Strengths
- Dr. Ritchie is an internationally recognized geriatrician with expertise in palliative care, home-based care, and health services research.
- Dr. Vranceanu is an accomplished psychologist with expertise in mindfulness and compassion interventions and virtual clinical trials.
- The PIs have forged a strong collaboration in the relatively short period of time that Dr. Ritchie has been at MGH. They have complementary skillsets, and have a track record of co-mentorship, NIH grants, and publications.

Weaknesses
- No significant weaknesses noted, but two of the Biosketches (Drs. Eric Macklin and Shelley Adler) are not tailored to this particular application; it is not clear how these investigators fit into the team.
- It is unclear why a sub-award is needed for an activity that does not require specialized knowledge and skills, especially given the resources at MGH.

3. Innovation:
Strengths
- Recruiting geographically diverse ADRD caregivers from across the US, including those in remote areas, is innovative.

Weaknesses
- The intervention, research design, assessment approach, and conceptual framework are not innovative.

4. Approach:
Strengths
- The applicants draw from the NIH Stage Model and principles from the Science of Behavior Change to develop, optimize, and test a behavioral intervention (minor).

- The plan to enroll >38% racial and ethnic minorities and geographically diverse caregivers from across the US, including those in remote areas, are strengths (moderate).
- The web-based platform will be available to caregivers after the program ends to facilitate skill development (minor).

Weaknesses

- Longer term implementation and sustainability of a recruitment approach that relies exclusively on a cut score on the Perceived Stress Scale (PSS) is questionable. It is not clear whether the PSS is routinely administered in practices, hospitals, non-governmental organizations, or community-based organizations that would refer caregivers to trialists or interventionists who wish to deploy this intervention in the future (major).
- Laudable efforts to focus on diversity and inclusion are partially undermined by the systematic exclusion of Spanish-speaking caregivers (major).
- It is unusual to have seven intervention targets in experimental therapeutics trials. The seven intervention targets appear arbitrary and interchangeable, but experimental therapeutics demands specificity. Moreover, most assessments of intervention targets rely exclusively on self-report, and most conflate state effects and trait effects, making them poorly suited for use in experimental therapeutics work (major).
- There are two very different pathways into trial enrollment: 1) resource-rich academic health centers in Massachusetts, and 2) nationally through social medial and the National Alliance for Caregiving. It is not clear whether the applicants have considered the potential for recruitment pathways to be imbalanced across study arms (e.g., greater levels of stress in MGH recruits, more severe dementia in national recruits, etc. (moderate).
- Given the prevalence of depression (60%, per the applicants), and that interventions for depression are reimbursable, while interventions for perceived stress are not, it is unclear why perceived stress is the outcome variable. This, too, could hinder longer term implementation and sustainment (moderate).
- It is unclear how behavioral management skills can be assessed without in vivo observation of skill use, or, at minimum self-report, using something like ecological momentary assessment (moderate)
- Participants must score above a cut score on the PSS in order to be eligible for inclusion, because “including caregivers who would not benefit is unethical.” If the applicants applied the same logic to, for example, loneliness and social support, they would exclude participants who did not meet a pre-specified criterion on these putative targets because it would be unethical to include participants who could not demonstrably improve on social support or loneliness. This is a conceptual weakness (moderate).
- It is unclear how psychotropic medications will be handled (minor).
- The definition of an adequate intervention dose is not provided (minor).
- The power analysis assumes independent observations, but the observations are not independent (minor).

5. Environment:

Strengths

- MGH is a well-resourced environment.

Weaknesses

- None noted.

Study Timeline:

Strengths

- Appropriate.

Weaknesses

- None noted.

Protections for Human Subjects:
Acceptable Risks and/or Adequate Protections.

Data and Safety Monitoring Plan (Applicable for Clinical Trials Only):
Acceptable.

Inclusion Plans:
- Sex/Gender: Distribution justified scientifically.
- Race/Ethnicity: Distribution justified scientifically.
- Inclusion/Exclusion Based on Age: Distribution justified scientifically.

Budget and Period of Support:
Recommend as Requested.

CRITIQUE 3:

Significance: 3
Investigator(s): 3
Innovation: 4
Approach: 5
Environment: 1

Overall Impact:
The proposed work develops an intervention to decrease stress by targeting compassion, mindfulness, and behavior management for dementia caregivers. This is an important area for intervention, as stress contributes to a host of physical and mental ailments among caregivers. The PIs have extensive experience with pragmatic trials, and the NIH Stage Model is used appropriately to describe the proposed work. However, as presented, it is not clear that the project will exert a sustained, powerful influence on the research field. The investigators have not provided rigorous evidence for the effects of the intervention components on the proposed health outcome (stress) or the target mechanisms. The mechanistic targets are not clear, and the investigators do not leverage a design which might help elucidate which aspects of the intervention are affecting which mechanistic targets. The thoughtful approach to diversity and inclusion is appreciated, and the feasibility and acceptability benchmarks are clear.

1. Significance:
Strengths
- The project addresses an important topic in the field – stress among dementia caregivers, which negatively impacts physical and mental health.
- If successful, this study would result in a group-based, virtual intervention that improves caregiver compassion, mindfulness, and behavioral management competencies. The next step would be a Stage III trial to determine its efficacy for stress and other health outcomes.

Weaknesses
- The prior research that serves as key support for this work is not strong. The investigators state "Our preliminary data shows that caregivers of persons with ADRD desire real-time guidance and support that weaves together emotional regulation, self-compassion, and behavioral management skills to successfully navigate the stress of the caregiving experience." Unfortunately, the reference provided is not preliminary data supporting this claim. The investigators also do not provide evidence for the efficacy of behavioral management or self-compassion interventions on caregiver stress. Some evidence for mindfulness-based interventions is provided.

2. Investigator(s):
Strengths
- The PIs are well-suited to this project. Dr. Ritchie has expertise in dementia care, including behavioral symptoms of dementia, and pragmatic trials for persons living with dementia. She directs the Dementia Care Collaborative, which will be used for subject recruitment. Dr. Vranceanu is leading two grants focused on building resiliency in caregiving dyads. She also has expertise in developing web platforms for two active NIH-funded projects. The investigators have complementary expertise, and have demonstrated an ongoing record of accomplishments that have advanced their field.

Weaknesses
- It does not appear that either PI has expertise in compassion or loneliness.

3. Innovation:
Strengths
- The applications seeks to shift current behavioral intervention research using the NIH Stage Model.
- The innovative elements of this application are virtual delivery of a shortened mindfulness intervention, tailoring of the intervention to dementia caregivers, and a strong focus on diversity and inclusion.

Weaknesses
- The components of the intervention are not novel, but the combination of elements and the tailoring of the intervention for dementia caregivers is innovative.

4. Approach:
Strengths
- The approach provides justification for the selected trial elements in the protocol synopsis. The overview of the proposed study design includes the stage of intervention research (stage 1A for development of intervention and open pilot; stage 1B for the pilot randomized clinical trial), and a rationale for the selected stage.
- The investigators have protocols for improving and monitoring intervention fidelity. The MPI will lead two half-day sessions training with the entire team before the trial begins. Clinicians will use session checklists to ensure consistency. The investigator team will meet weekly. All sessions will be recorded, and up to 20% of recordings will be reviewed by Dr. Adler.
- The study design includes discussion of how to reduce potential biases in recruitment and retention. The PIs will train study staff to engage in training around cultural competence and internalized racism to ensure interactions with patients are able to be rooted in trust and respect. The PIs have expertise in these strategies, as they have conducted similar training in prior studies.
- The omission of the skill-based training from the Health Education Program controls is appropriate.
- The inclusion/exclusion criteria are appropriate for the aim of maximizing internal validity

Weaknesses
- The mechanisms of action are not clear. The program combines mindfulness skills, compassion skills, and behavioral skills. The mechanistic targets should be measures of compassion, mindfulness, and behavioral management. However, the investigators also include loneliness, social support, caregiver self-efficacy, and relationship quality. Those are important concepts/competencies to capture in the model, but the intervention was not designed to act on them directly.
- Because the intervention combines several different strategies, it is difficult to disentangle which component of the intervention affects which mechanism. A factorial design or a more formal

mediation analysis might better explain the underlying relationships between the interventions and mechanisms.
- The investigators do not provide evidence for the efficacy of behavioral management or self-compassion interventions on caregiver stress. Some evidence for mindfulness-based interventions is provided.
- The investigators correctly state, “Traditional power analyses are also not appropriate for activities within NIH stage 1A, where the goal is to ‘explore’ feasibility and mechanistic target engagement for the sole purpose of refining the intervention and protocol.” However, they then say that they “will have 80% power to meet all the markers if the true rate of therapist fidelity is 95% and the true rate for all other benchmarks is 83%.” The power calculations should be based on benchmarks of 70%, as that is the passing criteria. At a minimum, a range of benchmarks should be used.

5. Environment:
Strengths
- The scientific environment at MGH and the Department of Medicine will contribute to the probability of success. The institutional support, equipment, and other physical resources are adequate.
- Dr. Ritchie directs the Dementia Care Collaborative, which will be used for subject recruitment. They also have partnerships with the National Alliance for Caregiving and the Massachusetts Alzheimer’s Disease Research Center.

Weaknesses
- None noted.

Study Timeline:
Strengths
- The study timeline describes start-up activities, anticipated rate of enrollment, and planned follow-up assessments.

Weaknesses
- The timeline is a bit aggressive in Year 1. The investigators are working with the Institutional Revie Board, developing scripts, and training staff at the same time that they are recruiting.
- Potential challenges and corresponding solutions are not discussed.

Protections for Human Subjects:
Acceptable Risks and/or Adequate Protections.
- This study poses minimal risk.

Data and Safety Monitoring Plan (Applicable for Clinical Trials Only):
Acceptable.
- The Data and Safety Monitoring Plan is clear and appropriate.

Inclusion Plans:
- Sex/Gender: Distribution justified scientifically.
- Race/Ethnicity: Distribution justified scientifically.
- Inclusion/Exclusion Based on Age: Distribution justified scientifically.

Resource Sharing Plans:
Acceptable.
- Plans to disseminate findings to academicians, community stakeholders, and participants.

Budget and Period of Support:
Recommend as Requested.

THE FOLLOWING SECTIONS WERE PREPARED BY THE SCIENTIFIC REVIEW OFFICER TO SUMMARIZE THE OUTCOME OF DISCUSSIONS OF THE REVIEW COMMITTEE, OR REVIEWERS' WRITTEN CRITIQUES, ON THE FOLLOWING ISSUES:

PROTECTION OF HUMAN SUBJECTS: ACCEPTABLE. Acceptable Risks and/or Adequate Protections are described in the application.

INCLUSION OF WOMEN PLAN: ACCEPTABLE. Of the 120 human subjects, 74% are women.

INCLUSION OF MINORITIES PLAN: ACCEPTABLE. Of the 120 human subjects, 13% are underrepresented minorities.

INCLUSION ACROSS THE LIFESPAN: ACCEPTABLE. The target population for this study is adults and older adults.

COMMITTEE BUDGET RECOMMENDATIONS: The budget was recommended as requested.

Footnotes for 1 R01 AG078204-01; PI Name: Ritchie, Christine S

NIH has modified its policy regarding the receipt of resubmissions (amended applications).See Guide Notice NOT-OD-18-197 at https://grants.nih.gov/grants/guide/notice-files/NOT-OD-18-197.html. The impact/priority score is calculated after discussion of an application by averaging the overall scores (1-9) given by all voting reviewers on the committee and multiplying by 10. The criterion scores are submitted prior to the meeting by the individual reviewers assigned to an application, and are not discussed specifically at the review meeting or calculated into the overall impact score. Some applications also receive a percentile ranking. For details on the review process, see http://grants.nih.gov/grants/peer_review_process.htm#scoring.

MEETING ROSTER

The roster for this review meeting is displayed as an aggregated roster that includes reviewers from multiple AG Special Emphasis Panels of the NIA SEP Aggregate roster for May 2022 Council for the 2022/05 council round.
This roster for AG is available here.
